# Versatile, vigilance, and gut microbiome support the priority of high-ranking hens

**DOI:** 10.3389/fvets.2023.1324937

**Published:** 2023-12-21

**Authors:** Zhijiang Xie, Limin Xing, Mengqiao Zhao, Lei Zhao, Jinling Liu, Yushan Li, Jiankang Gan, Siyu Chen, Hua Li

**Affiliations:** ^1^Guangdong Provincial Key Laboratory of Animal Molecular Design and Precise Breeding, Key Laboratory of Animal Molecular Design and Precise Breeding of Guangdong Higher Education Institutes, School of Life Science and Engineering, Foshan University, Foshan, China; ^2^Guangdong Tinoo’s Foods Group Co., Ltd., Qingyuan, China

**Keywords:** social hierarchy, hens, production performance, vigilance, gut microbiome

## Abstract

Dominance hierarchy exists in social animals and shows profound impacts on animals’ survival, physical and mental health, and reproductive success. Aggressive interaction, as the main indicator used to calculate social hierarchy, however, is not found in some female animals. In this study, we aimed to figure out the establishment of social hierarchy in hens that almost perform aggressive behaviors and investigated the interactions of social hierarchy with production performance and gut microbiome. Forty 49-day-old Qingyuan hens were randomly divided into four groups. The social hierarchy of hens was calculated by the relative position around the feeder. The rank 1 (R1), R2, R3, R4, R5, R6, R7, R8, R9, and R10 birds were determined in ascending order. Then, R1 and R2 birds (four duplicates, *n* = 8) were named as the high-ranking hens (HR) group, while R9 and R10 individuals were named as the low-ranking hens (LR) group (four duplicates, *n* = 8). The heart index (*p* = 0.01), number of visits per day, daily feed intake, and occupation time per day were higher in the HR group than LR group, but the LR group had a higher feed intake per visit than the HR group. The alpha diversity was significantly lower in the HR group than the LR group (*p* = 0.05). The relative abundance of phylum *Firmicutes* was higher while that of phylum *Deferribacterota* was lower in the HR group than LR group (*p* < 0.05). At the genus level, the relative abundance of *Succinatimonas*, *Eubacterium hallii group*, and *Anaerostipes* were higher in HR group than in LR group. The relative abundance of *Bacteroides*, *Mucispirillum*, *Subdoligranulum*, and *Barnesiellaceae unclassified* was higher in the LR group than HR group (*p* < 0.05). In conclusion, the rank of hens could be calculated by the relative position around the feeder when they compete for food. The dominant hens have a versatile. Moreover, they are more vigilant and have priority when foraging. Low-ranking hens adopt strategies to get enough food to sustain themselves. Hens of high-rank possess beneficial bacteria that use favorable substances to maintain the balance of the gut environment.

## Introduction

Social dominance is a ubiquitous phenomenon, which is among social creatures from insects to primates (1, 2). Social rank affects the distribution of dominant resources among individuals, and individuals with high rank tend to select areas where dominant resources are densely distributed, thus obtaining more relative resources. For example, cattle with high rank spend more time eating grains while grazing, and cattle with low rank spend more time circling along the pasture (3). The establishment of a social hierarchy is conducive to avoiding unnecessary conflicts, fights, and resource competition within groups. Low-ranking rhesus monkeys avoid attacks and threats from the dominant monkeys by “playing dumb” (4). The dominant individuals reproduce preferentially (5), which is essential for population development as well as the improvement of interspecific competitiveness (6).

Up to date, increased studies conducted on the social hierarchy have been also reported in chickens. It was already known that the highest-ranking rooster had priority for feeding (7) and crowing (8). Additionally, high-ranking roosters had higher plasma testosterone concentrations and lower ejaculatory testosterone compared to low-ranking individuals, suggesting that the social order in chickens has a significant impact on their hormonal regulation (9). Dominant hens force subdominant hens to produce submissive behavior through threat or force (10), and higher-rank hens had heavier eggs than lower-rank hens (11). This was because low-ranking hens were pecked at by the dominant hens, which caused them to stay in the nest for a shorter period and therefore tend to delay laying eggs (12). Notably, in the above-mentioned studies, aggressive interaction was the only indicator used to calculate the social hierarchy. However, there were rare aggressive behaviors in the group of some female animals. Thus, how to calculate the social hierarchy of these animals attracts our great attention.

In recent years, gut microbiota has become one of the major topics because of its association with the health and disease of the host. In chickens, a healthy microorganism balance is maintained within the gut (13), and complex functional interactions between intestinal microbes and host immunity are important for intestinal health (14). Our previous study has found that the health of the dominant roosters appears to benefit from short-chain fatty acids activity, while that of subdominant roosters may benefit from microbial function (15). However, little is known about the interaction between the social hierarchy of individuals with the production performance and gut microbiota in hens.

Thus, we will implement a precision feeding system to monitor the production performance of individuals. Accordingly, in this study, we aimed to figure out the establishment of social hierarchy in hens that almost perform aggressive behaviors, and investigate the interactions of social hierarchy with production performance and gut microbiome. Understanding these interactions would give new insights into strategies for enhancing hen health and well-being, as well as the improvement of management strategies for animals.

## Materials and methods

### Animals and feeding

Forty Qingyuan hens provided by Guangdong Tiannong Food Co., Ltd., at the age of 49 days with an average body weight of 652.5 g (SD = 50.12) were randomly selected for the study. Then 40 hens were randomly divided into four groups, with ten hens in each group. They were housed in a separate barn (1.5 m × 1.5 m × 2.5 m) with concrete ground with no litter material. All the experimental animals were tagged using colored leg rings and given free access to water and a commercial diet that was provided daily at 8:00 a.m.

### Establishment of social rank

Ten-minute feeding competition tests for hens in each group were performed at 57–59, and 76–78 days of age, a total of 6 times. Birds were deprived of food but not water from 18:00 the day before each test. Each separated barn contained a feeder, which only allowed two to four birds at a time to feed. Since aggressive behaviors were rare among these animals, a four-grade evaluation system was used for the relative position of foraging behavior at 30-s intervals for each bird. A score of 3 means hens were consuming the feed; score 2 means hens were not feeding but were within 2 to 6 cm of the feeder or had great motivation to approach the feeder; a score of score 1 means hens were squeezed out by the hens with score 2, or wandering around and seeking chances to approach the feeder; and a score of 0 means hens were independent and not fighting for food, or were over 20 cm away from the, or showed no desire to approach the feeder as they were foraging in the opposite direction of the feeder. The higher the score, the higher the social rank. The social rank of each hen was calculated for each test, and the final ranking was based on the sorted sum of the six evaluations. The higher the “sorted sum,” the lower the social rank. Subsequently, the rank 1 (R1), R2, R3, R4, R5, R6, R7, R8, R9, and R10 birds were determined in ascending order according to social rank. Then, R1 and R2 birds (four duplicates, *n* = 8) were named as the HR group, while R9 and R10 individuals were named as the LR group (four duplicates, *n* = 8).

### Feeding behavior, production performance, and organ index

From 67 days of age, a precise feeding system (9WJJ-20 produced by Guangdong Guangxing Animal Husbandry Equipment Co.) was introduced into the experiment, which allows only one bird at a time to feed. Each bird wore an ankle bracelet on its feet for system identification. By using the system, data including body weight, duration of feeding time, number of visits per day, occupation time per visit, feed intake per visit, occupation time per day, and daily feed intake were automatically recorded. Accordingly, the initial body weight, final body weight, and average daily weight gain were calculated during the whole experimental period. At the age of 91 days, all hens were humanely euthanized, and the bursa of the fabricius, heart, liver, and spleen of each bird was collected for weighing immediately. Organ index (%) = (organ weight/body weight) x 100% was used to determine the index for these organs.

### Vigilance test

At 61, 75, 79, 82, and 87 days of age, the same arena was used to measure the attentiveness of the birds in each group in response to a predator. The birds were accustomed to living worms (200 g/d) as a highly prized food source before the test, and they received them alongside regular meals from days 49 to 55. Hens were not provided with food and water at 18.00 on the day before the test. Regular feed was provided in one corner of the testing area, and regular feed containing live worms was placed in the other corner, 50 cm vertically above the feed, along with a hawk model (length: 30 cm; width: 30 cm). Additionally, during the 12-min test, hawk vocalizations were played three times (at 4, 8, and 12 min), and the hens’ response was graded on a scale from 0 to 2, with 0 denoting the lowest level of fear. Briefly, score 0 meant that there was no discernible change in the hen’s behavior; score 1 meant that the hen raised its head once and immediately went back to explore or eat; score 2 meant that the hen raised its head once and made an alarm call or walked quickly for more than 3 s or stopped moving for 3 to 10 s.

### Gut microbiome

On the slaughter day, the cecum contents of HR and LR birds were collected. Before being processed, they were kept in dry ice and then at-80°C. In order to create a library for sequencing, cecal DNA was extracted using the QIAamp DNA stool micro kit (Tiangen Biotech, Beijing, China) in accordance with the manufacturer’s instructions. Then, DNA quality was evaluated using agarose gel electrophoresis, and DNA quantity was determined using a UV spectrophotometer. 16S rRNA gene fragments containing the V3 and V4 hypervariable regions were amplified using primers 5’-CCTACGGGNBGCASCAG-3′ and 5’-GACTACNVGGGTATCTAATCC-3′ (16). The Illumina NovaSeq platform was used to sequence a total of 16S rRNA amplicons. QIIME2 (Version 2022–2) (17) was used to preprocess, quality filter, trim, denoise, merge, model, and analyze sequences through DADA2. The SILVA database was used to annotate feature sequences for each typical sequence, and the R package (v3.5.2) was used to graph the results. The concept of ASVs (Amplicon Sequence Variants) was used to construct classes of OTUs (Operational Taxonomic Units). Bioinformatics analysis was done using the ASV data to determine the relative abundance of taxonomic ranks and alpha diversity. Alpha diversity in terms of observed_otus, Shannon, Simpson, chao1, and Pielou_e indexes were used to explore within-group sample diversity. Higher index numbers represent higher alpha diversity. Utilizing the OmicStudio tools found at https://www.omicstudio.cn, a clustering correlation heatmap with indications was created.

### Statistical analysis

All data were analyzed by SPSS 25 and displayed as mean ± standard error (SE). Production performance data were checked for normality and homogeneity of variance, transformed where necessary, and analyzed by t-test. The Wilcoxon test was used to analyze the score obtained in the vigilance test. All values with *p* < 0.05 were considered statistically significant (Table 1).

**Table 1:** Organ index of birds between high-and low-ranking hens.

Organ index (g/kg)	HR	LR	SEM	*p*-value
Bursa	1.25 ± 0.56	1.26 ± 0.73	0.33	0.96
Heart	4.82 ± 0.71	4.03 ± 0.29	0.27	0.01
Liver	16.71 ± 1.15	18.40 ± 8.32	2.97	0.58
Spleen	1.73 ± 0.22	2.38 ± 2.01	0.71	0.38

HR means high-ranking hens, while LR means low-ranking hens.

### Ethical statement

This study was approved by the Animal Care Committee of Foshan University (Approval ID: FOSU#121).

## Results

### Social rank

**Figure 1:**
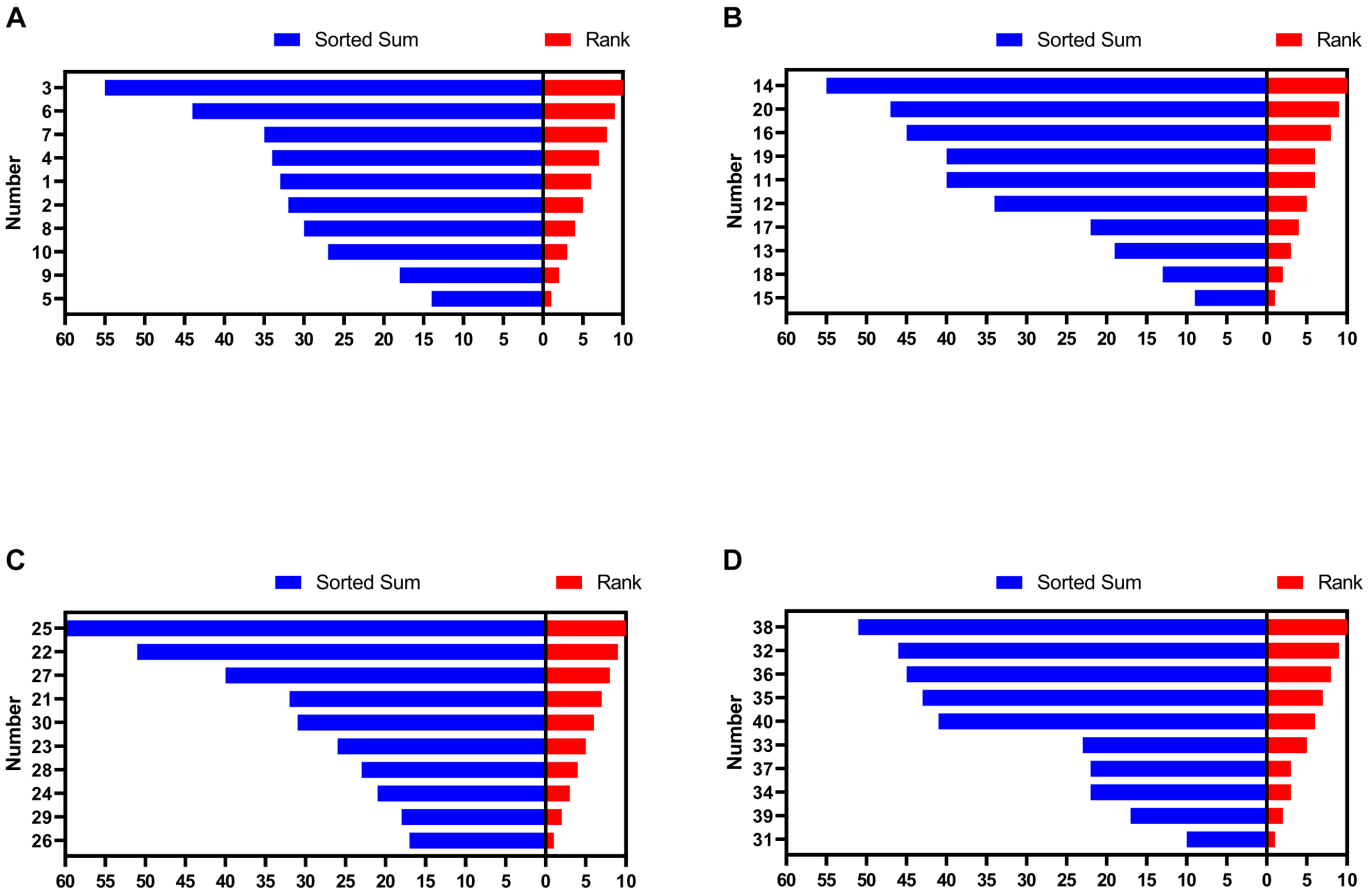
Social rank of hens in each group. **(A)** Group 1. **(B)** Group 2. **(C)** Group 3. **(D)** Group 4. The numbers on the y-axis represent each hen. HR means high-ranking hens, while LR means low-ranking hens.

As shown in Figure 1, hens numbered 5 (sorted sum: 14), 9 (18), 15 (9), 18 (13), 26 (17), 29 (18), 31 (10), and 39 (17) were in HR group and hens numbered 3 (sorted sum: 44), 6 (55), 14 (55), 20 (47), 22 (51), 25 (60), 32 (46), and 38 (51) were in LR group.

### Feeding behavior, production performance, and organ index

**Figure 2:**
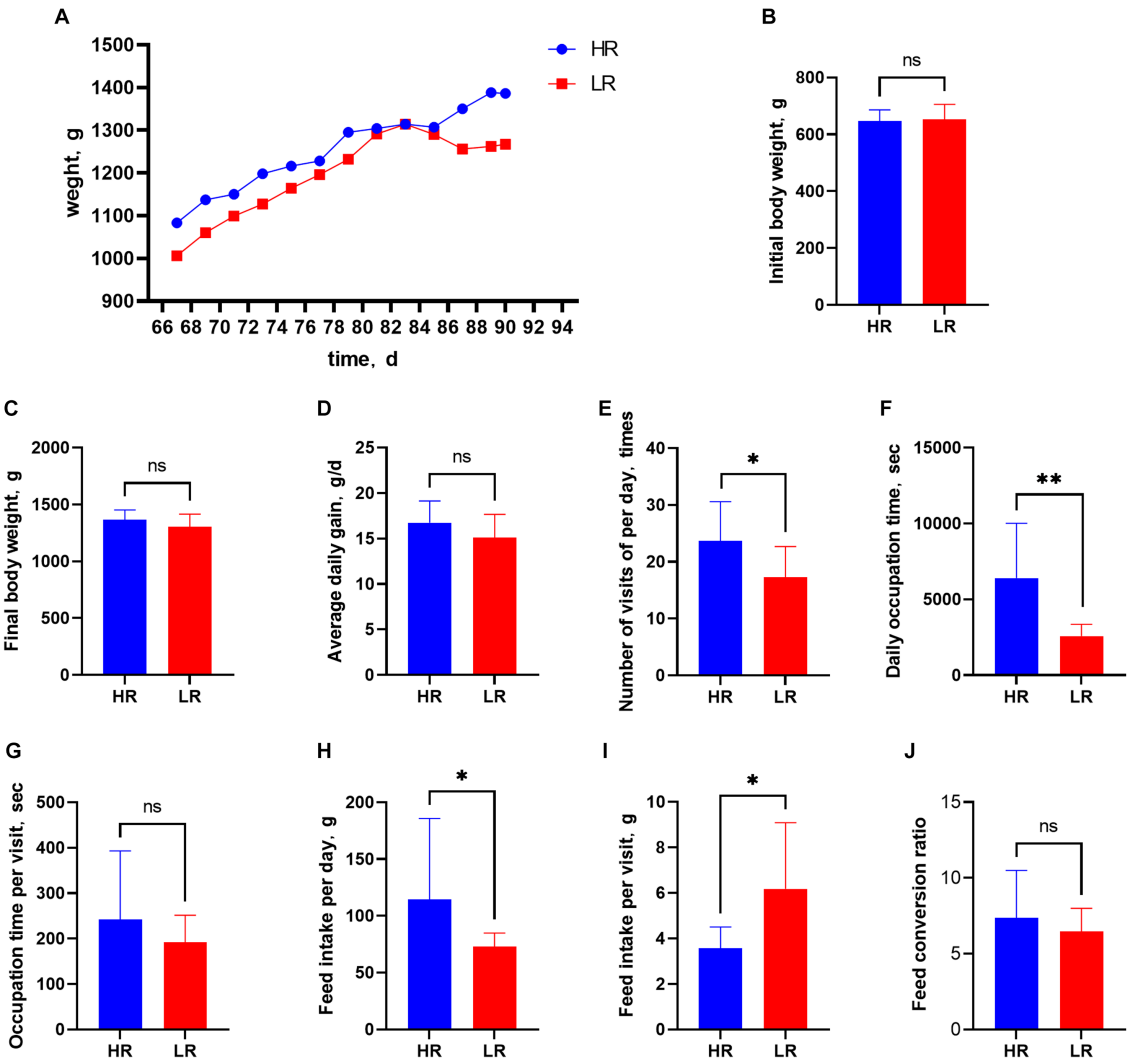
Feeding behavior and production performance. Figure 2 Feeding behavior and production performance. **(A)** Body weight from 67 to 90 days of age. **(B)** Initial body weight. **(C)** Final body weight. **(D)** Average daily gain. **(E)** Number of visits per day. **(F)** Occupation time per day. **(G)** Occupation time per visit. **(H)** Daily feed intake. **(I)** Feed intake per visit. **(J)** Feed conversion ratio. HR means high-ranking hens, while LR means low-ranking hens. “ns” means not significant. Asterisks denote significant deviations (*p < 0.05, **p < 0.01).

The body weight of all animals increased gradually from 67 to 90 days of age, but that of the HR group was consistently heavier than that of the LR group (Figure 2A). Initial body weight (Figure 2B), final body weight (Figure 2C), average daily gain (Figure 2D), occupation time per visit (Figure 2G), and feed conversion ratio (Figure 2J), were not significantly different between HR and LR hens. Number of visits per day (Figure 2E; *p* < 0.05), daily feed intake (Figure 2H; *p* < 0.05), and occupation time per day (Figure 2F; *p* < 0.01) were significantly higher, while feed intake per visit (Figure 2I; *p* < 0.05) was significantly lower in the HR than LR. The heart index was significantly higher in the HR group than in the LR group (Table 2; *p* = 0.01), but there was no significant difference between the two groups in the bursal, liver, and spleen indexes.

**Table 2:** Vigilance tests.

	0	1	2	X^2^	*p*-value
HR	0 (0%)	0 (0%)	8 (100%)	7.27	0.03
LR	1 (12.5%)	4 (50%)	3 (37.5%)		

The fear score in response to the vigilance test of high-and low-ranking hens. HSR means high-ranking hens, while LSR means low-ranking hens.

### Vigilance tests

In response to the vigilance test (Table 2), score 2 of the HR group was (100%) significantly higher than that of the LR group (50%).

### Gut microbiome

**Figure 3:**
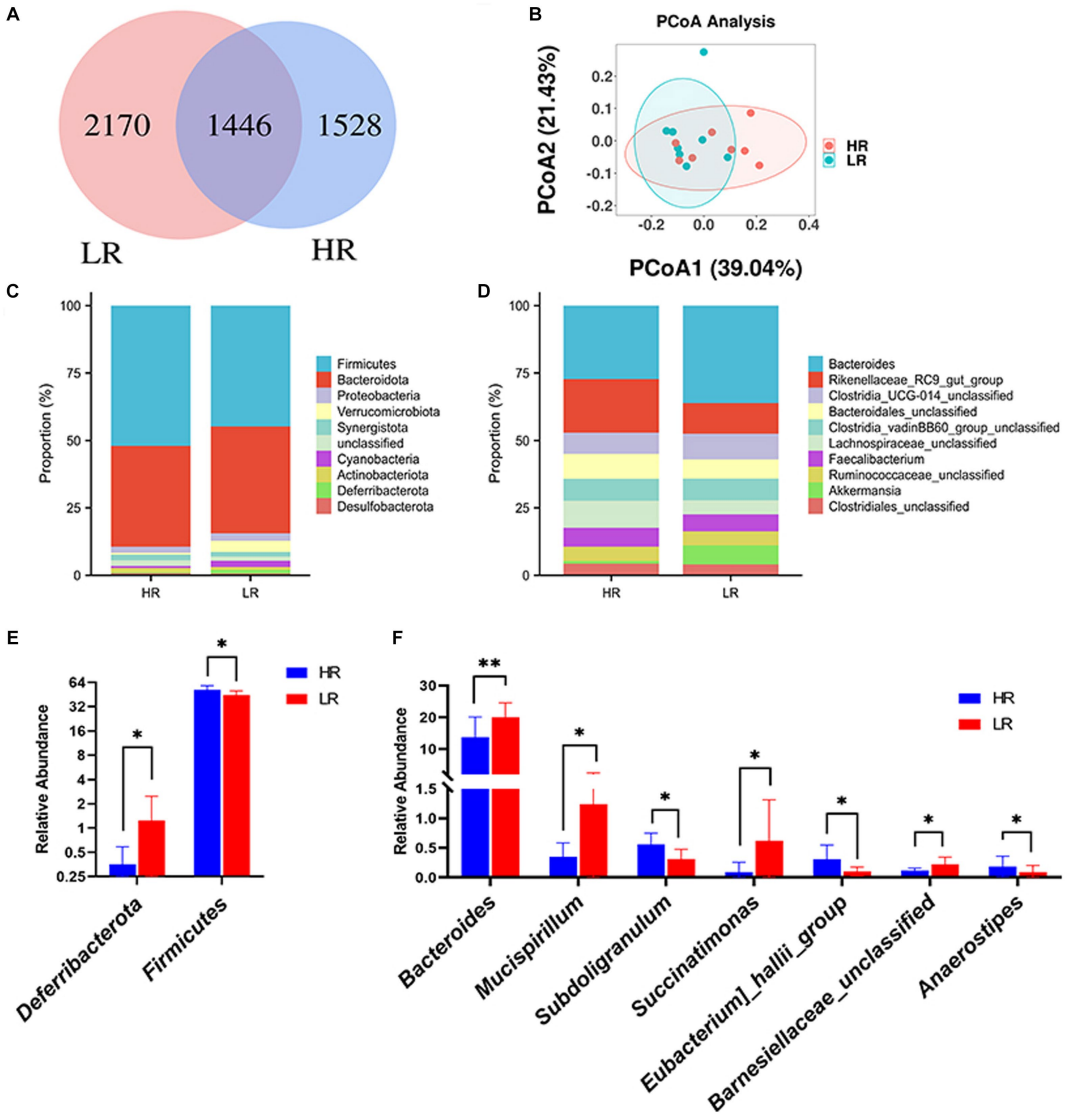
Gut microbiome. **(A)** ASV distribution Venn diagram. **(B)** Principal coordinate analysis (PCOA) of the cecal microbiota. **(C)** Relative abundance of gut microbes at phylum level. **(D)** Relative abundance of gut microbes at genus level. **(E)** Differential analysis of gut microbes at phylum level. **(F)** Differential analysis of gut microbes at the genus level. HR means high-ranking hens, while LR means low-ranking hens. Asterisks denote significant deviations (*p < 0.05, **p < 0.01).

In our analysis, a total of 5,144 ASVs were observed across both HR and low-risk LR groups. Among them, 1,528 ASVs were exclusively present in the HR group, while 2,170 ASVs were unique to the LR group (Figure 3A). At the phylum level, the top three phyla in the chicken cecum were *Bacteroidetes*, *Firmicutes*, and *Proteobacteria* (Figure 3C). The relative abundance of *Firmicutes* (*p* < 0.05) was higher in high-ranking hens than in low-ranking hens, while the relative abundance of *Deferribacterota* (*p* < 0.05) was lower in high-ranking hens than in low-ranking hens (Figure 3E). At the genus level, the top three microbes were *Bacteroides*, *Rikenellaceae RC9 gut group*, and *Clostridia UCG-014 unclassified* (Figure 3D). The relative abundance of *Succinatimonas* (*p* < 0.05), *Eubacterium hallii group* (*p* < 0.05), and *Anaerostipes* (*p* < 0.05) in the high-ranking hens was higher than that of low-ranking hens. At the same time, the relative abundance of *Bacteroides* (*p* < 0.01), *Mucispirillum* (*p* < 0.05), *Subdoligranulum* (*p* < 0.05), and *Barnesiellaceae unclassified* (*p* < 0.05) was lower in high-ranking hens than in low-ranking hens (Figure 3F).

The beta diversity of the gut microbiome between HR and LR hens was significantly different by PCoA analysis based on the weighted unifrac distance matrix (Figure 3B). Alpha diversity was significantly lower in the HR group than in the LR group in terms of observed_otus and chao1 index (Table 3; *p* = 0.05), with no difference in terms of the Shannon, Simpson, and Pielou indexes.

**Table 3:** Alpha diversity.

Items	HR	LR	SEM	*p*-value
Observed_otus	774.50 ± 178.30	924.10 ± 86.08	70.00	0.05
Shannon	7.58 ± 0.52	7.71 ± 0.53	0.26	0.64
Simpson	0.98 ± 0.01	0.98 ± 0.03	0.01	0.46
Chao1	777.20 ± 179.80	930.70 ± 86.41	70.52	0.05
Pielou_e	0.79 ± 0.03	0.78 ± 0.05	0.02	0.58

Alpha diversity of gut microbiome between high-and low-ranking hens. HR means high-ranking hens, while LR means low-ranking hens.

**Figure 4:**
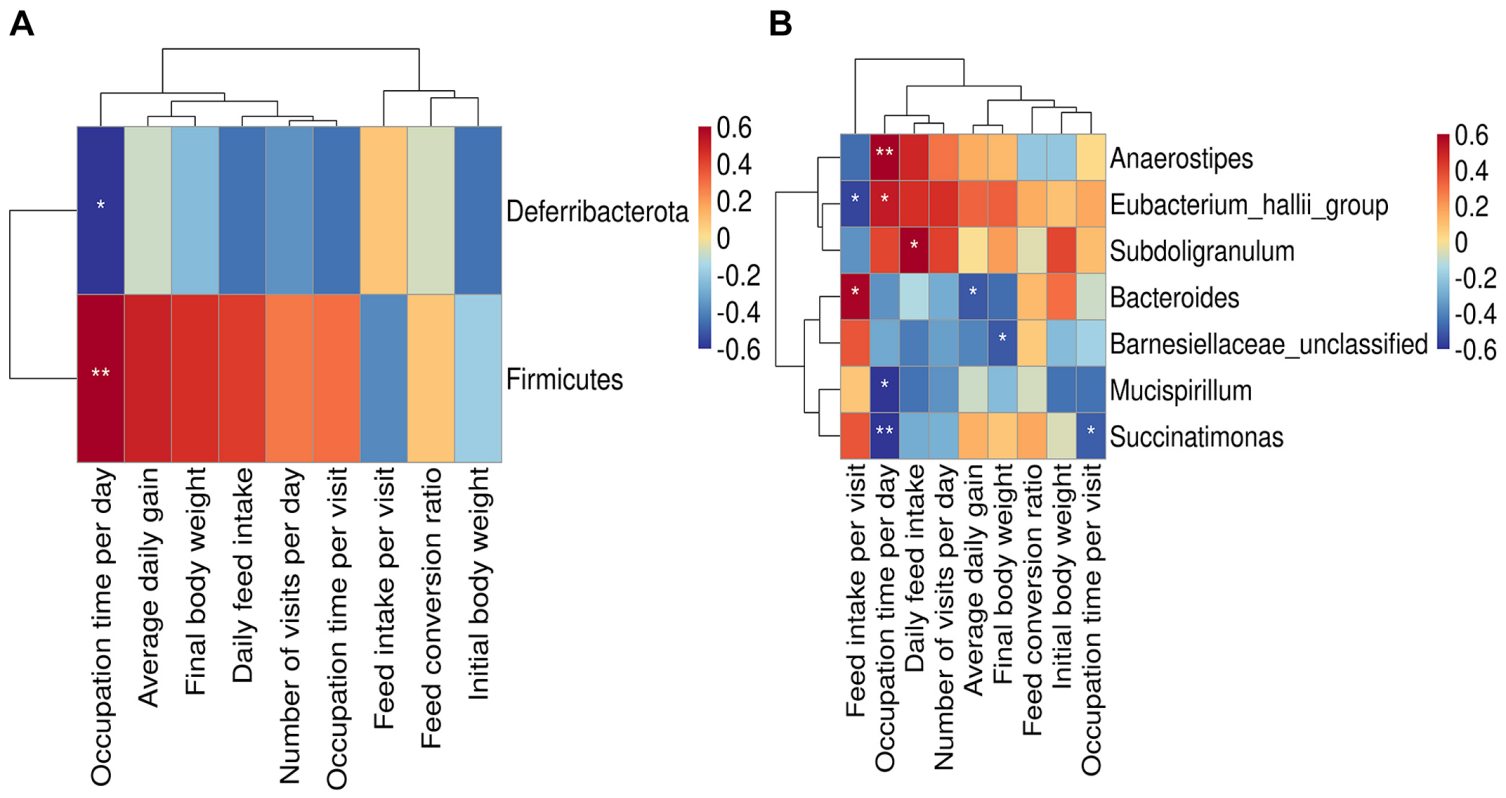
Relationship between bacteria and feeding behavior or production performance. **(A)** Heat map of the correlation between gut microbes at phylum level and feeding behavior or productive performance. **(B)** Heat map of the correlation between gut microbes at genus level and feeding behavior or productive performance. Asterisks denote significant deviations (*p < 0.05, **p < 0.01).

The relative abundance of phylum_*Firmicutes* was significantly and positively correlated with occupation time per day (*p* < 0.01), and that of phylum *Deferribacterota* was significantly and negatively correlated with occupation time per day (*p* < 0.05) (Figure 4A). At the genus level (Figure 4B), the relative abundance of *Anaerostipes* was significantly and positively correlated with occupation time per day (*p* < 0.01). The relative abundance of *Eubacterium hallii group* was significantly and positively correlated with feed intake per visit (*p* < 0.05) and negatively correlated with occupation time per day (*p* < 0.05). *Subdoligranulum* was significantly and positively correlated with daily feed intake (*p* < 0.05). The relative abundance of *Bacteroides* significantly and positively correlated with feed intake per visit (*p* < 0.05), and negatively correlated with average daily gain (*p* < 0.05). The relative abundance of *Barnesiellaceae unclassified* was significantly and negatively correlated with final body weight (*p* < 0.05). The relative abundance of *Mucispirillum* and *Succinatimonas* was significantly negatively correlated with occupation time per day (*p* < 0.05).

## Discussions

The social hierarchy is usually established in terms of aggressive interactions in both the male and female animals as mentioned above (7, 10). However, in some cases aggressive behavior is rare in female animals, which makes the establishment of a social hierarchy difficult to identify. In this study, we set up food competition and found a clear order of access to food resources among the Qingyuan partridge hens. The higher-ranking birds had priority to forage food, the subdominant birds always observed and then searched for opportunities to forage, and the low-ranking birds were not involved in the food competition or surrendered to higher-rank birds. Accordingly, we used the relative position around the feeder to calculate the social rank. The principle of advantageous rank benefit suggests that social rank affects the distribution of advantageous resources among individuals, and higher-ranking individuals are prone to have more advantageous resources (18). For example, Shimmura et al. (19) found the results of the competition for food resources were positively correlated with the social rank of hens, and high-ranking hens had priority to use welfare facilities such as perches and laying boxes (20). In this study, the number of visits per day as well as the occupation time per day in the HR group was significantly higher than that in the LR group, indicating that hens in higher social rank had more opportunities to feed. However, initial body weight, final body weight, and average daily gain were not different between high-and low-ranking hens. This is consistent with a pig study (21) and may be explained by the fact that animals in the same environment will ensure their own required intake by selecting multiple equally effective feeding strategies (22). Pigs will adopt different dietary patterns of meat eaters (few long meals every day) or nibblers (many short meals every day). We found out that chickens of high-ranking had feeding priority (7) while low-ranking chickens also had access to food to satisfy their needs after the higher rank birds finished feeding. Feed intake per visit was significantly higher in the LR group than in the HR group, which was unexpected and may suggest that hens with low social rank need to meet their energy and nutritional requirements within a limited period of time. This is probably the main reason why no difference in body weight between the high-and low-ranking hens, regardless of the consistently higher body weight of HR than LR. Our results show that hens of high social rank have a tremendous advantage in terms of access to resources. While, hens of lower rank use a strategy of increasing feed intake per visit to ensure daily food intake.

In a previous study, better immune functions were found in higher-rank rhesus monkeys than in lower individuals (23). We calculated organ indexes related to immune function and did not find the difference in liver, spleen, and bursa indexes between high-and low-ranking birds. Additionally, the heart index of the hens in the HR group was significantly higher than that in the LR group, which may suggest better aerobic capacity in HR birds than in LR birds (24). Notably, greater exercise capacity means more energy consumption, that is to say, the high-ranking hens may get more food but also consume more energy, which may be one of the causes for no differences in body weight between high-and low-ranking hens.

Anti-predator behavior refers to a range of behaviors exhibited by prey species in response to predators, including alertness, aggression, and vocalization (25). Low-ranking avians are reported to be more vigilant when they take the form of visual scanning (26). Stress is a manifestation of hypervigilance and is considered to be a response to uncertainty (27). Therefore, low-ranking animals are deemed to be more fearful and suffer more stress as compared to high-ranking animals (28). Nevertheless, high-ranking hens showed more vigilance in the face of danger than low-ranking hens in this study. As known, an appropriate level of stress is considered an adaptive coping mechanism because it allows animals to avoid potential threats and seize opportunities to improve ranking or access resources (29). We speculated that the higher vigilance of high-ranking hens may be due to perceived threats from outside.

The alpha diversity is mainly used to reflect species richness and as a stressor indicator reported in chicken (30), mice (31), and dairy studies (32). In the present study, the alpha diversity of low-ranking hens was higher than those of high-ranking hens. This finding may suggest the higher stress of higher-ranking birds than lower-ranking birds, which is somehow supported by the fear response between HR and LR hens. At the phylum level, *Bacteroidetes, Firmicutes*, and *Proteobacteria* were the most abundant in the cecal microbiota, which was consistent with previous studies (33, 34). *Firmicutes* play a pivotal role in the fermentation of complex polysaccharides (35) and contribute significantly to the digestion of plant fibers (36). Their higher abundance in the gastrointestinal tract aids in providing growing animals with essential nutrients and energy resources (37). Additionally, *Firmicutes* encompass a substantial number of gram-positive bacteria, some of which are beneficial and contribute to pathogenic defense while maintaining a balance in intestinal microflora (38). *Deferribacteres*, another bacterial group, obtain energy through exclusive or parthenogenetic anaerobic metabolism, influencing iron metabolism and being associated with intestinal iron homeostasis (39). Microorganisms need iron for growth and metabolism (40), and thus the lower abundance of *Deferribacteres* may be one of the reasons for the lower alpha diversity in high-ranking hens than in low-ranking hens. Genus *Succinatimonas* can utilize small amounts of sugar and produce succinic and acetic acids from glucose as the primary fermentation end products (41). A more prosperous abundance of the intestinal genus *Eubacterium hallii* represents a better level of social function (42), which can use glucose, fermentation intermediates acetate, and lactate to create the beneficial butyrate for intestinal health (43, 44). *Anaerostipes* are known for their ability to produce butyrate from sugars as well as lactate and acetate (45), which has a potentially beneficial role in promoting host health (46). *Bacteroides* are anaerobic, bile-resistant, non-spore-forming gram-positive rods that are clinically important pathogens seen in most anaerobic infections (47). In the cecum samples of pigs, *Bacteroides* were negatively associated with fatness traits (48). Besides, the reduction of *Mucispirillum* abundance in gut microbiota was known to be linked with decreased blood triglycerides and free fatty acids in mouse (44). There is evidence that *subdoligranulum* is associated with chronic inflammation and inefficient metabolic control (45). *Barnesiellaceae unclassified* was significantly and positively correlated with propionic acid in mice (46). These results may suggest more beneficial bacteria and better intestinal environment in high ranking than low ranking hens.

The relative abundance of *Firmicutes*, *Deferribacterota*, *Anaerostipes*, *Eubacterium hallii group*, *Mucispirillum*, and *Succinatimonas* were correlated with occupation time per day. The magnitude of occupation time per day represents the appetite of individuals. Appetite is an inherent drive required to support organic existence (49). Feed intake per visit and occupation time per visit depend on the degree of stomach fullness and gastric capacity of individual hens. In our study, feed intake per visit was related to the relative abundance of *Eubacterium hallii group* and *Bacteroides*, while occupation time per visit was related to the relative abundance of *Succinatimonas*. In cattle, feed intake depends mainly on appetite and rumen capacity (50). Whether these bacteria have any effect on appetite is not yet known, and further research is needed to figure out this interaction between appetite and above gut microbes, so as to better understand the microbial function on production performance.

## Conclusion

The social hierarchy of hens that rarely express aggressive interaction could be calculated by the relative position around the feeder when they compete for food. The dominant hens have an versatile, are more vigilant, and have priority access to food resources, while low-ranking hens adopt strategies to get enough food to sustain themselves, and therefore show no differences in body weight with high-ranking hens. Hens of high rank possess beneficial bacteria that use favorable substances to maintain gut health. The relative abundance of some bacteria is found to be associated with appetite; however, their underlying mechanism should be further classified.

## Data availability statement

The datasets presented in this study can be found in online repositories. The names of the repository/repositories and accession number(s) can be found at: https://www.ncbi.nlm.nih.gov/, PRJNA1029363.

## Ethics statement

The animal studies were approved by Institutional Animal Care and Use Committee of Foshan University. The studies were conducted in accordance with the local legislation and institutional requirements. Written informed consent was obtained from the owners for the participation of their animals in this study.

## Author contributions

ZX: Data curation, Formal analysis, Investigation, Methodology, Writing – original draft, Writing – review & editing. LX: Data curation, Formal analysis, Investigation, Methodology, Writing – review & editing. MZ: Data curation, Investigation, Methodology, Writing – review & editing. LZ: Data curation, Investigation, Methodology, Writing – review & editing. JL: Methodology, Writing – review & editing. YL: Methodology, Writing – review & editing. JG: Data curation, Resources, Writing – review & editing. SC: Conceptualization, Methodology, Project administration, Writing – original draft, Writing – review & editing. HL: Conceptualization, Writing – review & editing.
